# Research on Control Method of the Power System of Stepping-Type Anchoring Equipment

**DOI:** 10.3390/s21217123

**Published:** 2021-10-27

**Authors:** Guoyong Su, Yongcun Guo, Pengyu Wang, Gang Cheng, Dongyang Zhao

**Affiliations:** State Key Laboratory of Mining Response and Disaster Prevention and Control in Deep Coal Mines, Anhui University of Science and Technology, Huainan 232001, China; Guoyongs005@sina.cn (G.S.); ycguo2018@163.com (Y.G.); chgmech@mail.ustc.edu.cn (G.C.); ZDY17364321329@163.com (D.Z.)

**Keywords:** anchoring equipment, control method, pump-controlled hydraulic system, sliding mode control

## Abstract

To improve the roadway adaptability and control accuracy of anchoring equipment, a stepping anchoring device was designed. A permanent-magnet synchronous motor control and a harmonic suppression algorithm were integrated to optimize the dynamic control system of stepping-type anchoring equipment. The results of an experimental simulation and analysis showed that when the coefficient of coal rock hardness *f* = 5, 6, and 7, the pulsation coefficient of the hydraulic pump outlet pressure, hydraulic motor output speed, and pump-controlled hydraulic cylinder advance speed in the hydraulic circuit of a pump-controlled motor did not exceed 3% after the equipment based on sliding mode control (SMC) entered the steady state, while the maximum pulsation coefficient was only 32.5% of the PI control. Based on the SMC, the harmonic components of the permanent magnet synchronous motor in the power system were suppressed and compensated for. This enhanced the stiffness of the hydraulic system under motor drive. When the rock stiffness factor gradually changed from *f* = 5 to *f* = 8 and increased suddenly from *f* = 5 to *f* = 6, the pressure overshoot at the outlet of the hydraulic pump of the pump-controlled motor system was reduced from 11.19% to 7.97% and from 61.19% to 52.88%, respectively, compared with that before the optimization. It was thereby proven that SMC based on harmonic suppression can effectively reduce the system pulsation caused by the multi-factor coupling of anchoring equipment and provide technical support for the optimal control of the power system of stepping-type anchoring equipment.

## 1. Introduction

Anchoring operation is an important task in coal mining. The high rate of occurrence of geological hazards in underground coal seams and strong contingency make this operation very challenging. According to statistics [1,2], roadway support operations account for more than 60% of the total drilling time. Therefore, the improvement of the reliability and work efficiency of anchor support operations will directly enhance the speed and efficiency of roadway excavation.

Stable and efficient electro-hydraulic power system is the foundation and guarantee for improving the operation efficiency of mining equipment [3,4,5]. At present, there are many kinds of electro-hydraulic control methods [6,7,8], mainly including valve control mode based on Intelligent PI control [9,10], intelligent PID control [11,12], SMC [13,14,15,16], and pump control mode based on variable speed driver technology [17,18]. Among the anchoring equipment [19,20,21], machine-mounted rotary hydraulic roof bolters are the most widely used. The electro-hydraulic system in such equipment often employs the Ziegler-Nichols algorithm to tune the PID parameters. Although this control method is simple and easy to implement, the parameter adjustment is more complicated and the control accuracy of hydraulic systems with low damping, strong nonlinearity, and time-varying parameters is not high [22,23]. Ahn et al. [24] designed a set of adaptive robust force controllers and used the gradient descent method to adjust the controller parameters to improve the stability of the hydraulic system under time-varying parameters and large external load fluctuations. However, this system has disadvantages, such as a poor low-speed stability, limited speed adjustment range, slow response, and high requirements for oil quality. Wang [25] proposed a hydraulic system control strategy for a roof bolter based on a variable-frequency pump-controlled hydraulic drive system and PID parameter tuning with an intelligent fusion optimization algorithm. Simulations and experiments showed that under different working conditions, the roof bolter can apply intelligent fusion optimization algorithms to adjust the working parameters of the equipment automatically according to the changes in the peak oil pressure of the buffer chamber of the variable stroke mechanism. This ensures that the roof bolter performs well under reasonable parameter-matching working conditions and that the equipment functions at maximum power. Hoang [26] analyzed the influence of system parameter uncertainty and external interference on the control of the system, and put forward a new control strategy for the control of hybrid fluid power equipment based on the fundamentals of the fast terminal sliding mode, which ensured that the tracking error converges to the origin in a limited time.

In the foundation of the analysis and research above, a variety of intelligent algorithms were applied to optimize and tune the PID control parameters in the valve control system or pump-control system of the anchoring equipment to improve the control accuracy and stability of the equipment. Since the pump-control hydraulic system is driven by a frequency-conversion permanent magnet synchronous motor (PMSM), the controller, PMSM, hydraulic pump, etc., exhibit multivariable and nonlinear strong coupling effects [27,28,29], and the system pulses tend to be more complex.

In the present work, stepping-type anchoring equipment was designed. The overall coupling mechanism of the converter-PMSM, pump-controlled motor, and pump-controlled hydraulic cylinder was analyzed and a mathematical model of the drive control system of the walking anchoring equipment was constructed. Furthermore, a coordinated control algorithm for PMSM harmonic suppression compensation was proposed to actively suppress the pulsation of stepping-type anchoring equipment. 

## 2. Stepping-Type Anchoring Equipment and Power System Composition

**Figure 1:**
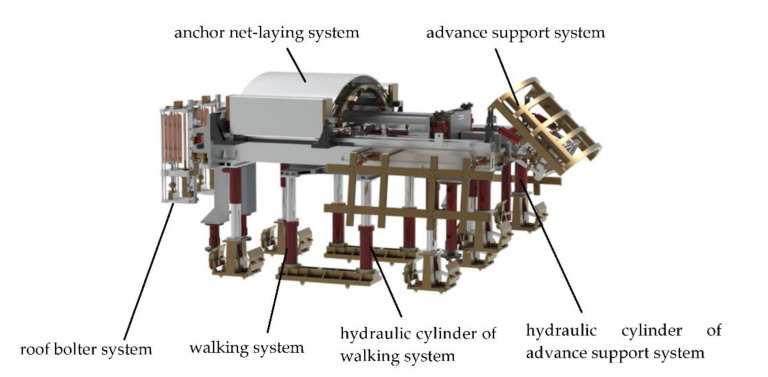
Stepping-type anchoring equipment.

The stepping-type anchoring equipment designed to improve the roadway adaptability of the anchoring equipment is shown in Figure 1. The equipment mainly comprises a mechanical system and a power system. The mechanical system comprises subsystems such as advance support system, anchor net-laying system, walking system, and a roof bolter system. The walking system uses hydraulic cylinders for walking forward and is used for the mobile transportation of anchoring equipment; the anchor net-laying system is used to complete the net-laying work before anchoring the fully mechanized excavation face. The power system comprises a hydraulic system and an electric and control system. The hydraulic system mainly includes a quantitative hydraulic pump, an electromagnetic reversing valve, a supporting column hydraulic cylinder, a quantitative motor and pushing cylinder, and other components. The electric and control system comprises a PMSM, a controller, and its controller. The working process of the stepping-type anchoring equipment is as follows: after the equipment reaches the designated working position, the hydraulic cylinder of the walking system is supported on the ground; meanwhile, the hydraulic cylinder of the advance support system is supported on the ground, and the anchor net-laying system is extended to spread the anchor net to the roof and sidewalls of the roadway. Finally, the anchoring system adjusts the posture of the bolter drill so that it can complete anchoring operations at different positions.

**Figure 2:**
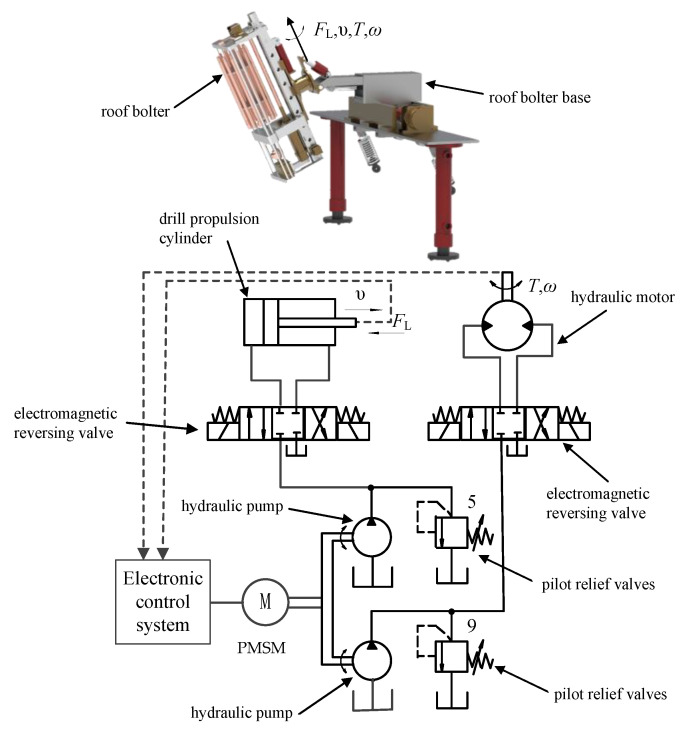
Control system of the stepping-type anchoring equipment.

To improve the ability of the stepping-type anchoring equipment to work in coal and rock roadways with different levels of hardness, the power system adopts a frequency-conversion electric motor to drive double pumps. Since the roof bolter system in this equipment is most affected by the hardness of the coal and rock, the power system of the anchoring equipment was simplified, considering the roof bolter as the main research object. The power system and control unit are shown in Figure 2. 

## 3. Mathematical Model of Stepping-Type Anchoring Equipment Power System

The optimal cutting speed and thrust speed of the roof bolter during the anchoring process of stepping-type anchoring equipment vary greatly with the hardness of the rock. In addition, there exists a strong coupling between the transient mechanical characteristics of the PMSM and the hydraulic load; any change in a parameter of either will affect the working status of the entire system. Hence, it is necessary to establish a unified control equation under the coupling action of multiple factors, such as those of thecontroller–PMSM–hydraulic system. The use of such a unified control equation is essential to provide a theoretical basis for optimizing the performance of the pump-controlled hydraulic system.

### 3.1. Mathematical Model of Permanent Magnet Synchronous Motor

To improve the control accuracy of the power system of stepping-type anchoring equipment, considering the distortion magnetic field of the permanent magnet-synchronous electric motor, the dead time of the inverter, and the tube voltage drop, a large number of harmonic components are introduced into the three-phase current of the main circuit [30,31]. Therefore, a mathematical model of PMSM with motor distortion magnetic field and a harmonic current was built [32,33] as follows:

(1) $$\left\{\begin{array}{c} \left[\begin{array}{c} u_{d}\\ u_{q} \end{array}\right]=\left[\begin{array}{cc} i_{d} & -\psi_{q}\\ i_{q} & \psi_{d} \end{array}\right]\left[\begin{array}{c} R\\ \omega_{e} \end{array}\right]+\frac{d}{dt}\left[\begin{array}{c} \psi_{d}\\ \psi_{q} \end{array}\right]\\ \left[\begin{array}{c} \psi_{d}\\ \psi_{q} \end{array}\right]=\left[\begin{array}{c} L_{d}i_{d}+\psi_{f1}-5\psi_{f5}\cos\left(-6\omega_{e}t+\theta_{f5}\right)+7\psi_{f7}\cos\left(6\omega_{e}t+\theta_{f7}\right)\ldots \\ 5\psi_{f5}\sin\left(-6\omega_{e}t+\theta_{f5}\right)+7\psi_{f7}\sin\left(6\omega_{e}t+\theta_{f7}\right)\ldots \end{array}\right]\\ \left[\begin{array}{c} i_{d}\\ i_{q} \end{array}\right]=\left[\begin{array}{c} i_{d1}+i_{5}\cos\left(-6\omega_{e}t+\theta_{5}\right)+i_{7}\cos\left(6\omega_{e}t+\theta_{7}\right)+\ldots \\ i_{q1}+i_{5}\cos\left(-6\omega_{e}t+\theta_{5}\right)+i_{7}\cos\left(6\omega_{e}t+\theta_{7}\right)+\ldots \end{array}\right]\\ \begin{array}{c} T_{e}=\frac{3p_{n}i_{q}}{2}\left[i_{d}\left(L_{d}-L_{q}\right)+\psi_{f}\right]\\ J_{T}\frac{d\omega_{P}}{dt}=T_{e}-B_{T}\omega_{P}-T_{L} \end{array} \end{array}\right.$$

Here, *ω*_e_ = *p*_n_*ω*_p_ where *u*_d_ and *u*_q_ are the two-phase rotating coordinate system *d-q* axis voltages (V); *R* is stator phase resistance (Ω); *i*_d_ and *i*_q_ are two-phase rotating coordinate system *d-q* axis currents (A); *ψ*_d_ and *ψ*_q_ are Flux linkage in the d-q rotating coordinate system (Wb); *ω*_e_ is the electrical angular velocity (rad/s); *L*_d_ and *L*_q_ are two-phase rotating coordinate system *d-q* axis inductances (H); *ψ*_f_ is the rotor flux linkage (Wb); *θ*_e_ is the rotor electrical angle (rad); *ω*_e_ is the electrical angular velocity (rad/s); *ω*_p_ is the mechanical angular velocity (rad/s); *p*_n_ is the number of pole pairs; *J*_T_ is the moment of inertia of the motor (kg·m^2^); *B*_T_ is the damping coefficient of the motor shaft (N·m·s/rad); *T*_e_ is the electromagnetic torque (N·m), and *T*_L_ is the PMSM load torque (N·m).

### 3.2. Mathematical Model of Hydraulic System and Its Load

To facilitate the control of the rotary torque and feed rate of the roof bolter and improve the stability of the system, a “one machine with two pumps” system was adopted as the power source. A theoretical model of the hydraulic motor, hydraulic cylinder and load was established, and the influence of multiple factors on the stability of the system was explored.

(2) $$\left\{\begin{array}{c} T_{\mathrm{pm}/\mathrm{pg}}=\frac{p_{\mathrm{pm}/\mathrm{pg}}D_{\mathrm{pm}/\mathrm{pg}}}{\eta_{\mathrm{pm}/\mathrm{pg}}}\\ Q_{\mathrm{pm}/\mathrm{pg}}=D_{\mathrm{pm}/\mathrm{pg}}\omega_{\mathrm{pm}/\mathrm{pg}}-p_{\mathrm{pm}/\mathrm{pg}}\lambda_{\mathrm{pm}/\mathrm{pg}}\\ Q_{\mathrm{pm}}-Q_{m}=\frac{V_{\mathrm{pm}}}{\beta_{e}}\frac{dp_{\mathrm{pm}}}{dt}\\ Q_{m}=D_{m}\omega_{m}+p_{\mathrm{pm}}\left(C_{\mathrm{pm}}+C_{m}\right)\\ J_{m}\frac{d\omega_{m}}{dt}=D_{m}p_{\mathrm{pm}}\eta_{\mathrm{mm}}-B_{m}\omega_{m}-T_{\mathrm{Lm}}\\ \frac{V_{\mathrm{pg}}}{\beta_{e}}\cdot \frac{dP_{\mathrm{pg}}}{dt}=D_{\mathrm{pg}}\omega_{\mathrm{pg}}-A\nu_{g}-P_{\mathrm{pg}}\left(C_{\mathrm{pg}}+C_{g}\right)\\ m_{d}\frac{d\nu_{g}}{dt}=P_{\mathrm{pg}}A-B_{g}\nu -F_{g} \end{array}\right.$$

where *T*_pm/pg_ is the input torque of the pump-controlled motor system and pump-controlled hydraulic cylinder system (N·m); *D*_pm/pg_ is the pump displacement of the pump-controlled motor system and pump-controlled hydraulic cylinder system (m^3^/rad); *p*_pm/pg_ is the outlet pressure of the pump-controlled motor system and pump-controlled hydraulic cylinder system (MPa); *η*_pm/pg_ is the mechanical efficiency of the pump-controlled motor system and pump-controlled hydraulic cylinder system; *Q*_pm/pg_ is the pump-controlled motor system and pump-controlled hydraulic output flow of the cylinder system pump (m^3^/s); *ω*_pm/pg_ is the pump speed of the pump-controlled motor system and pump-controlled hydraulic cylinder system (rad/s); *λ*_p_ is the leakage coefficient of the pump (m^3^); *Q*_m_ is the input flow of the hydraulic motor (m^3^/s) *V*_pm_ is the volume of the enclosed cavity between the pump and the hydraulic motor (m^3^); *V*_pm_ is the volume of the enclosed cavity between the pump and the hydraulic cylinder (m^3^); *β*_e_ is the elastic modulus of the oil volume in the enclosed cavity (N/m^2^); *D*_m_ is the displacement of the hydraulic motor (m^3^/rad); *ω*_m_ is the speed of the hydraulic motor (rad/s); *C*_pm/pg_ is the leakage coefficient of the pump-controlled motor system and pump-controlled hydraulic cylinder system; *C*_m_ is the leakage coefficient of the hydraulic motor; *C*_g_ is the leakage coefficient of the hydraulic cylinder; *J*_m_ is the equivalent moment of inertia of the hydraulic motor output shaft (kg·m^2^); *η*_mm_ is the mechanical efficiency of the hydraulic motor; *B*_m_ is the viscous damping coefficient of the hydraulic motor output shaft (N·m·s/rad); *T*_Lm_ is the hydraulic motor load Torque (N·m); *ν*_g_ is the feed speed of the hydraulic cylinder (m/s); *A* is the effective area of the hydraulic cylinder (m^2^); *m*_d_ is the equivalent mass of the hydraulic cylinder (kg); *B*_g_ is the damping coefficient of the hydraulic cylinder (N·m·s/rad), and *F*_g_ the is hydraulic cylinder load (N).

According to the drilling characteristics and rock breaking mechanism of hydraulic roof bolter, the drilling energy per unit volume of rock (i.e., the rock crushing work ratio), *E*/*V*, and the required drilling torque and propulsion for different rock hardness conditions can be expressed as follows [34,35]:

(3) $$\left\{\begin{array}{c} E_{1}=F_{g}\nu_{g}\Delta t\\ E_{2}=T_{\mathrm{Lm}}\omega_{m}\Delta t\\ E=E_{1}+E_{2}=F_{g}\nu_{g}\Delta t+T_{\mathrm{Lm}}\omega_{m}\Delta t=\left(F_{g}\nu_{g}+T_{\mathrm{Lm}}\omega_{m}\right)\Delta t\\ V=\nu_{g}\Delta tA_{Z}=\frac{\pi }{4}\nu_{g}\Delta tD_{Z}^{2}\\ \frac{E}{V}=\frac{F_{g}}{A_{Z}}+\frac{T_{\mathrm{Lm}}\omega_{m}}{\nu_{g}A_{Z}} \end{array}\right.$$

where *E*_1_ is the work done by the roof bolter to push the hydraulic cylinder within time Δ*t* (J); *E*_2_ is the work done by the roof bolter rotating the motor within time Δ*t* (J); *V* is the rock-breaking volume of the roof bolter within time Δ*t* (m^3^); *D*_z_ is the diameter of the borehole (m); *A*_z_ is the cross-sectional area of the borehole (m^2^), and A is the active area of the piston rod of the hydraulic propulsion cylinder (m^2^).

(4) $$\left\{\begin{array}{c} n_{m}=\frac{c}{f\sqrt{D}}\\ T_{\mathrm{Lm}}=\frac{\Delta \sigma \left(D^{2}-D0^{2}\right)}{8000}\\ F_{g}=\left(2.5∼4\right)Df\\ \nu_{g}=\frac{8T_{\mathrm{Lm}}n_{m}}{\left(D^{2}-D0^{2}\right)\sigma } \end{array}\right.$$

where *c* is the cutting speed constant; *f* is the rock’s hardness coefficient; *D* is the outside diameter of the drill (mm); *D*_0_ is the inner diameter of the drill (mm); “2.5~4” is the range of correction coefficients of the formula; *σ* is the compressive strength of the rock (MPa), and Δ is the limit of drilling (mm).

Equations (3) and (4) show that the values of the rock crushing work ratio *E*/*V* required when the hydraulic roof bolter drills into different rocks differ from the best hydraulic motor speed for drilling different rocks. This leads to corresponding differences in the pressure and flow of the hydraulic cylinder and hydraulic motor circuit, especially under working conditions wherein the hardness coefficients of soft and hard rocks differ greatly; these differences will have a greater impact on the stable operation of the motor. Therefore, it is necessary to study the control method used for the frequency-conversion hydraulic roof bolter.

## 4. Research on the Control Method of Power System of Stepping-Type Anchoring Equipment 

To improve the working ability of the stepping-type anchoring equipment in complex coal and rock roadways where there are large differences in the rock layer hardness, its power system adopts frequency-conversion vector control to ensure that there is a good matching relationship between the torque, speed, and rock hardness of the roof bolt.

### 4.1. PI Control of Pump-Controlled Hydraulic System

**Figure 3:**
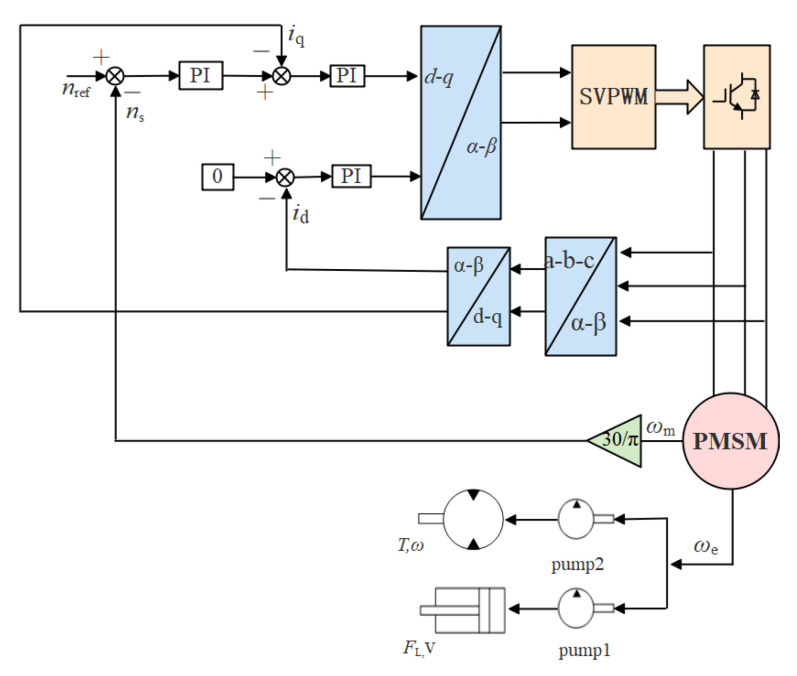
Functional block diagram of pump controlling the hydraulic system based on the CSPI.

The variable frequency vector control method can independently control the motor current to realize the decoupling of the magnetic field and the torque and can effectively improve the mechanical characteristics of PMSM. Hence, the variable frequency control system of the stepping-type anchoring equipment adopts PI as the speed control loop for characteristic research. The dynamic system based on the control system of PI (CSPI) is shown in Figure 3.

### 4.2. Sliding Mode Control of Pump-Controlled Hydraulic System

Linear controls such as PI control are easily affected by internal parameters and load fluctuations. In contrast, sliding mode control (SMC) is a discontinuous control strategy with time-varying switching characteristics. This process is unaffected by system parameters and external disturbances. The SMC of PMSM is described by [32]:

(5) $$\left\{\begin{array}{c} \begin{array}{c} x_{1}=\omega_{\mathrm{ref}}-\omega_{p}\\ x_{2}=-\omega_{{}_{p}}^{\;\;{\;}^{\prime }\;\;\;}\\ x_{{}_{1}}^{\;\;{\;}^{\prime }\;\;\;}=-\omega_{{}_{p}}^{\;\;{\;}^{\prime }\;\;\;}=\frac{1}{J_{T}}\left(T_{L}-\frac{3p_{n}\psi_{f}}{2}i_{q}\right)\\ x_{{}_{2}}^{{}^{\prime }}=-\omega_{{}_{p}}^{\;\;{\;}^{\prime }\;\;\;\;{\;}^{\prime }\;\;\;}=-\frac{3p_{n}\psi_{f}}{2J_{T}}i_{q} \end{array}\\ \begin{array}{c} i_{q}^{\;\;{\;}^{\prime }\;\;\;}=\frac{2J_{T}}{3p_{n}\psi_{f}}\left[cx_{2}+\varepsilon \left(s\right)+ks\right]\\ i_{q}^{*}=\frac{2J_{T}}{3p_{n}\psi_{f}}\int_{0}^{t}\left[cx_{2}+\varepsilon \left(s\right)+ks\right]dt \end{array} \end{array}\right.$$

where *ω*_ref_ is reference speed of motor (rad/s), *n*_ref_ = 30*ω*_ref_/π; *i*_q_* is the reference current of the *q*-axis (rad/s), and in the formula of the paper, ()’ is the derivative of (). 

In the paper, SMC is adopted as the system control method and the stability of vector control based on SMC is the basis of the stability of this control system. Therefore, the Lyapunov function is supplemented for stability analysis of the control system to demonstrate the validity and reliability of the content studied in this paper. The proof process is as follows [32]:

The sliding mode surface function is defined as:

(6) $$s=cx_{1}+x_{2}$$

Lyapunov function is defined as:

(7) $$V=\frac{s^{2}}{2}$$

Pursuant to Lyapunov’s stability decision theorem, the requisite conditions for system stability are *t* ≠ 0, V’ < 0, i.e., ss’ < 0:

(8) $$s^{{}^{\prime }}=cx_{1}^{{}^{\prime }}+x_{2}^{{}^{\prime }}$$

The paper employs the exponential convergence law method, which yields:

(9) $$s^{\;\;{\;}^{\prime }\;\;\;}=-\varepsilon \mathrm{sgn}\left(s\right)-ks\begin{array}{cc} & \varepsilon >0,k>0 \end{array}$$

(10) $$\begin{array}{cc} ss^{{}^{\prime }} & =s\left[-\varepsilon \mathrm{sgn}\left(s\right)-ks\right]\\ & =-\varepsilon \left|s\right|-ks^{2}<0 \end{array}$$

Hence, this control system is asymptotically stable.

**Figure 4:**
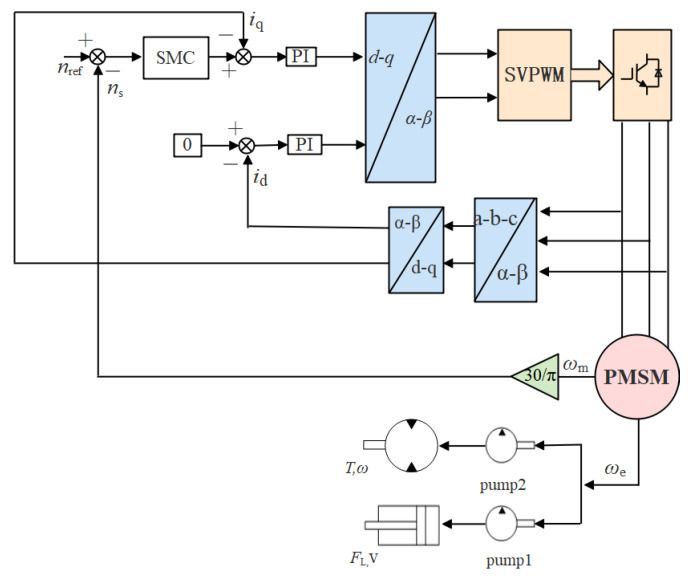
Functional block diagram of pump controlling the hydraulic system based on the CSSMC.

The dynamic system based on the control system of SMC (CSSMC) is shown in Figure 4.

### 4.3. Experimental Simulation and Analysis

To study the change law of the dynamic system during the anchoring operation of the stepping-type anchoring equipment and the control effect of the dynamic system based on the CSPI and CSSMC, MATLAB and AMESim were used for joint simulation. The simulation parameters are listed in Table 1.

**Figure 5:**
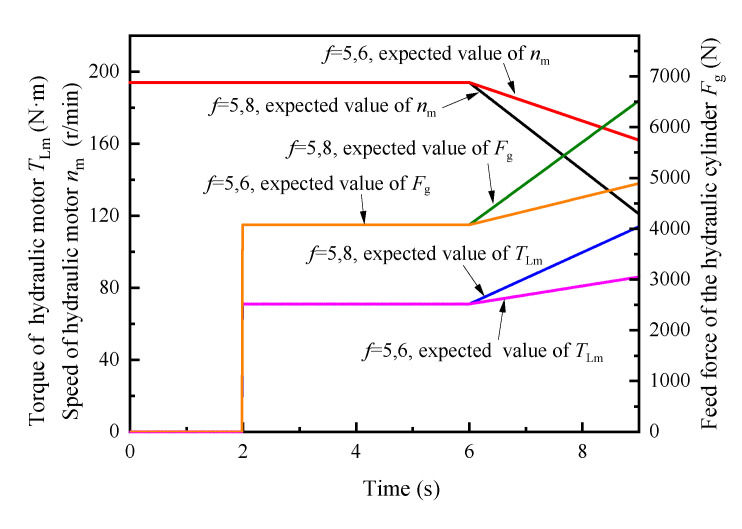
Analogue simulation input curve.

**Figure 6:**
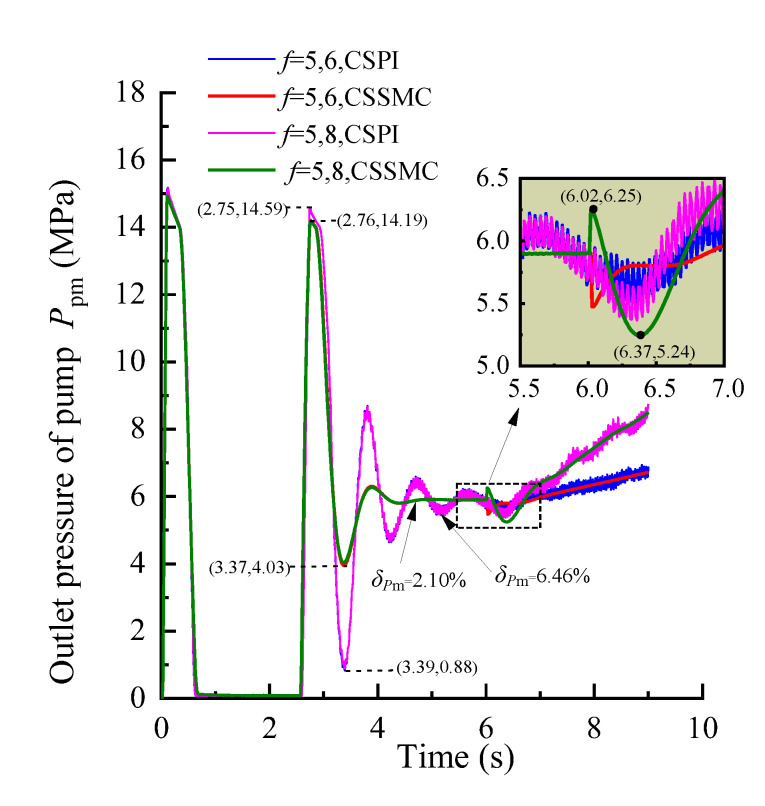
Variation curve of the hydraulic pump outlet pressure based on the CSPI and CSSMC.

**Figure 7:**
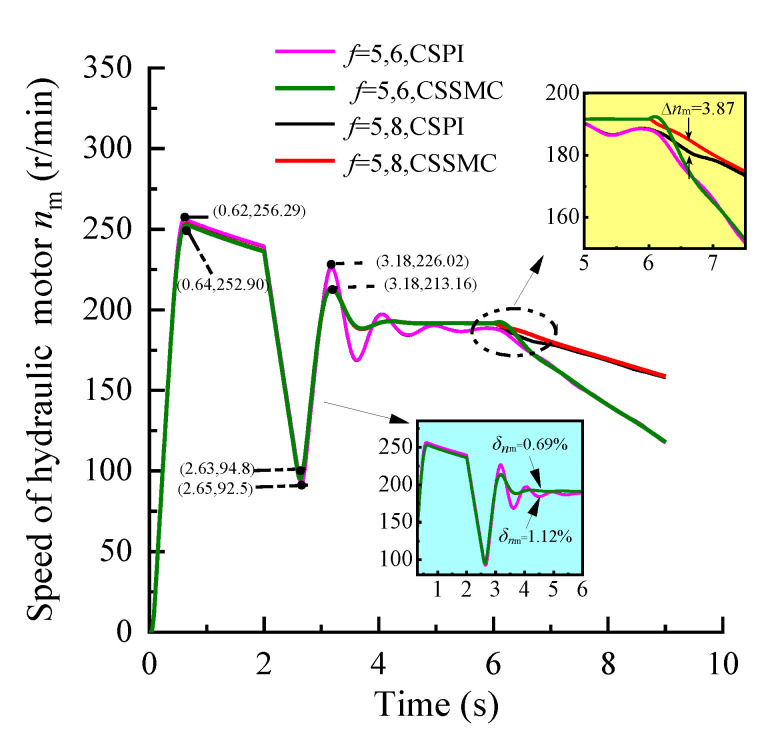
Variation curve of the hydraulic motor speed based on the CSPI and CSSMC.

**Figure 8:**
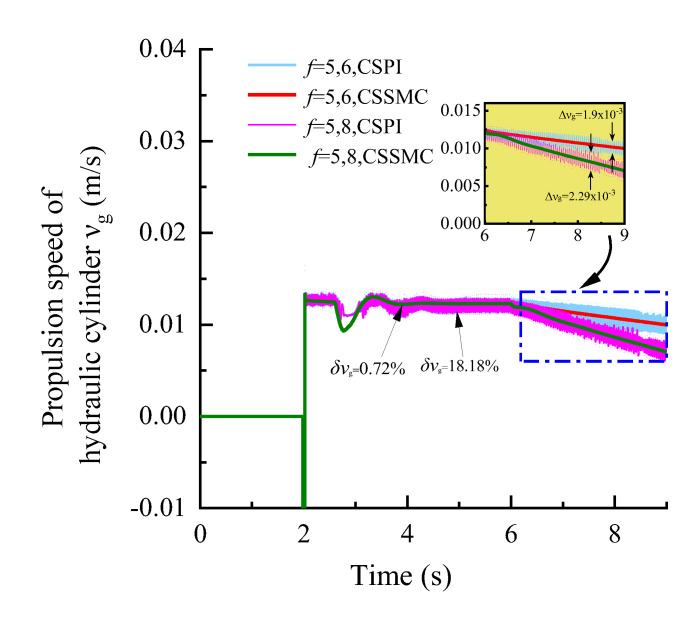
Variation curve of the hydraulic cylinder speed based on the CSPI and CSSMC.

The simulation conditions were set as: operating time *t* = 0–6 s, roadway rock hardness *f* = 5; *t* = 6–9 s, rock hardness *f* gradually increased from 5 to 6 and 8, respectively. According to Formula (4), the input curve of the simulation system, based on the expected torque and the expected speed of the hydraulic motor, and the expected feed force of the hydraulic cylinder is obtained, as shown in Figure 5. The experimental simulation results are shown in Figure 6, Figure 7, Figure 8.

The speed pulsation coefficient of the hydraulic motor is defined as *δ*_nm_ and the velocity pulsation coefficient of hydraulic cylinder is defined as *δ*_ng_:

(11) $$\delta_{{{}_{n}}_{{}_{{}_{m}}{/}_{{{}_{n}}_{{}_{{}_{g}}}}}}=\frac{x_{\max}-x_{\min}}{\bar{x}}$$

The pulsation coefficient of the outlet pressure pulsation is defined as follows *δ*_p_a__:

(12) $$\delta_{{{}_{P}}_{{}_{a}}}=\frac{x_{\max}-x_{\min}}{x_{\max}+x_{\min}}$$

where *x*_max_, *x*_min_, *x* are the maximum, minimum, and average values of the variables evaluated for pulsation.

Figure 6, Figure 7, Figure 8 show that, after applying a constant drive load (rock hardness coefficient *f* = 5), the hydraulic pump outlet pressure overshoot by the CSSMC is lower than by the CSPI. After the pressure, motor speed, and hydraulic cylinder propulsion speed enter a steady state, the pulsation coefficients are reduced from 6.46%, 1.12%, and 18.18% (by the CSPI) to 2.10%, 0.69%, and 0.72% (by the CSSMC). This finding suggests that the proposed CSSMC can improve the dynamic quality of the system and that the physical quantity of the system output is smoother compared with the traditional CSPI.

**Figure 9:**
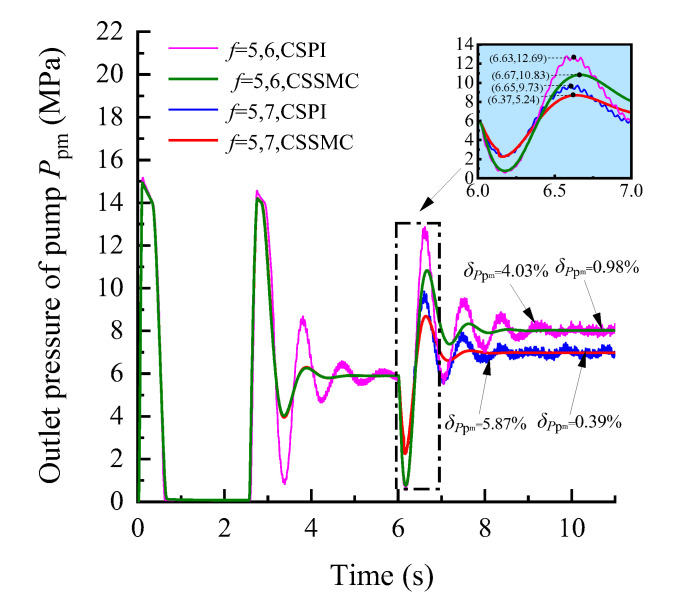
Variation curve of the hydraulic pump outlet pressure based on the CSPI and CSSMC.

**Figure 10:**
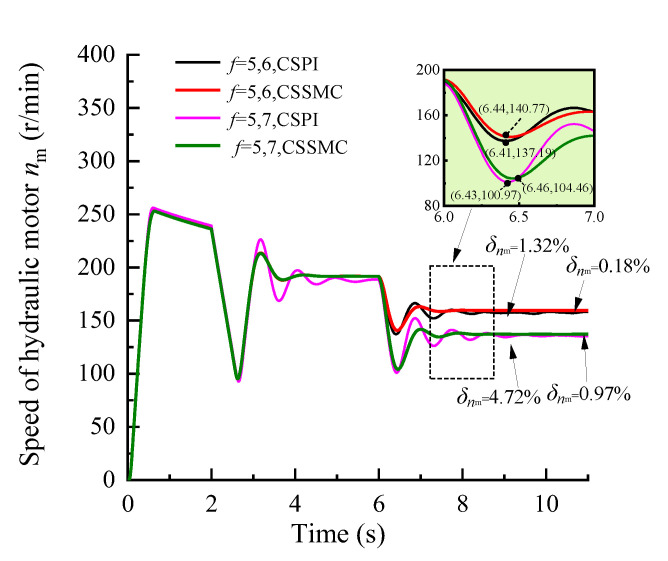
Variation curve of the hydraulic motor speed based on the CSPI and CSSMC.

**Figure 11:**
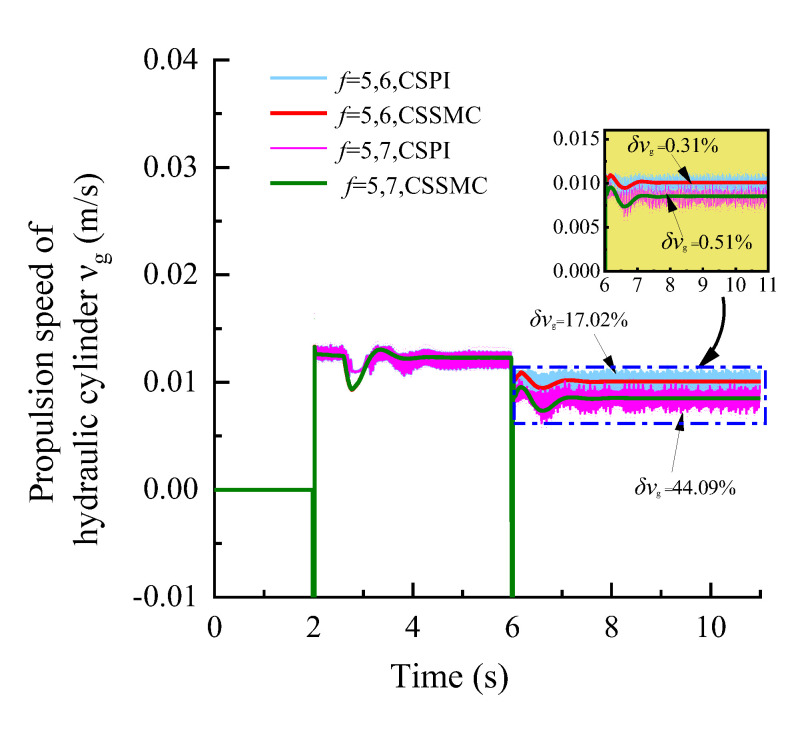
Variation curve of the hydraulic cylinder speed based on the CSPI and CSSMC.

To further study the performance of the hydraulic system based on the CSSMC, the hydraulic cylinder and hydraulic motor load of the anchoring equipment were used to provide a step signal input to simulate the operating conditions of the anchoring equipment under conditions of larger changes in the hardness of the rock layers: for *t* = 0–6 s, the roadway rock hardness is *f* = 5; for *t* = 6–11 s, the roadway rock hardness is *f* = 6–7. The change curves for outlet pressure of the hydraulic pump, the output speed of the hydraulic motor, and the forward speed of the hydraulic cylinder in the hydraulic circuit of the pump control motor are shown in Figure 9, Figure 10, Figure 11, respectively.

When the coefficient of rock hardness changes abruptly from *f* = 5 to *f* = 6, the hydraulic pump outlet pressure of the pump-controlled motor system based on the CSPI fluctuates the most, with an overshoot of 67.87%, while the pulsation coefficients of pressure, motor speed, and hydraulic cylinder propulsion speed after entering the steady state become 5.87%, 1.32%, and 17.02%, respectively. By the CSSMC, the hydraulic pump outlet pressure, the speed of hydraulic motor, and the speed of hydraulic cylinder are 0.39%, 0.18%, and 0.31%, respectively (see Figure 9, Figure 10, Figure 11). When the rock hardness coefficient is abruptly changed from *f* = 5 to *f* = 7, the parameters of the hydraulic control system change in a similar pattern, which will not be repeated here. To further compare and analyze the pulsation of each physical quantity after the system enters the steady state in Figure 6, Figure 7, Figure 8, Figure 9, Figure 10, Figure 11, the statistical results are shown in Table 2.

## 5. Performance Improvement Analysis of the Power Control System of Stepping-Type Anchoring Equipment

From Figure 6, Figure 7, Figure 8, Figure 9, Figure 10, Figure 11, it can be seen that the dynamic quality of the system can be improved based on the CSSMC compared with the CSPI. However, it was also found that under the condition of a sudden load change, the pulsation overshoot of the system is large, meaning that it is necessary to further optimize its control strategy to improve the kinetic energy stiffness of the hydraulic control power system.

### 5.1. Harmonic Suppression Compensation Coordinated Control of Permanent Magnet Synchronous Motor

According to the working characteristics of PMSM, the mechanical characteristics of PMSM periodically pulsated change due to the harmonic current of the converter, the tube resistance voltage drop, etc., while the pulsating component mainly exists in the form of the 5th and 7th harmonics [36,37]. Therefore, to realize the current control of the *d*-axis and *q*-axis of the 5th and 7th harmonics in the system, a synchronous rotating coordinate axis system corresponding to the 5th and 7th harmonics was established. The coordinate transformation matrix from the *d*-*q* coordinate system to 5*d*-*q* and 7*d*-*q* is thus as follows [36,37,38]:

(13) $$\left\{\begin{array}{c} T_{1dq\to 5dq}=\left[\begin{array}{cc} \cos(-6\theta ) & \sin(-6\theta )\\ -\sin(-6\theta ) & \cos(-6\theta ) \end{array}\right]\\ T_{1dq\to 7dq}=\left[\begin{array}{cc} \cos(6\theta ) & \sin(6\theta )\\ -\sin(6\theta ) & \cos(6\theta ) \end{array}\right] \end{array}\right.$$

By substituting Equation (9) into Equation (1), we obtain the following relation:

(14) $$\left[\begin{array}{c} u_{d}\\ u_{q} \end{array}\right]=\left\{\begin{array}{c} \begin{array}{c} 5dq:\\ \left[\begin{array}{c} \begin{array}{cc} \; & Ri_{1}\cos\left(6\omega t+\theta_{1}\right)-\omega L_{q}i_{1}\sin\left(6\omega t+\theta_{1}\right)+5\omega L_{q}i_{q5}\\ \; & +Ri_{d5}-7\omega L_{q}i_{7}\sin\left(12\omega t+\theta_{7}\right)+Ri_{7}\cos\left(12\omega t+\theta_{7}\right)+6\omega L_{d}i_{q5} \end{array}\\ \begin{array}{cc} \; & Ri_{1}\sin\left(6\omega t+\theta_{1}\right)+\omega L_{d}i_{1}\cos\left(6\omega t+\theta_{1}\right)-5\omega L_{d}i_{d5}\\ \; & +Ri_{q5}+7\omega L_{d}i_{7}\cos\left(12\omega t+\theta_{7}\right)+Ri_{7}\sin\left(12\omega t+\theta_{7}\right)-6\omega L_{q}i_{d5} \end{array} \end{array}\right] \end{array}\\ \begin{array}{c} 7dq:\\ \left[\begin{array}{c} \begin{array}{cc} \; & Ri_{1}\cos\left(-6\omega t+\theta_{1}\right)-\omega L_{q}i_{1}\sin\left(-6\omega t+\theta_{1}\right)+5\omega L_{q}i_{5}\sin\left(-12\omega t+\theta_{5}\right)\\ \; & +Ri_{5}\cos\left(-12\omega t+\theta_{5}\right)-7\omega L_{q}i_{q7}+Ri_{7d}-6\omega L_{d}i_{7}+\ldots \end{array}\\ \begin{array}{cc} \; & Ri_{1}\sin\left(-6\omega t+\theta_{1}\right)+\omega L_{d}i_{1}\cos\left(-6\omega t+\theta_{1}\right)-5\omega L_{d}i_{5}\cos\left(-12\omega t+\theta_{5}\right)\\ \; & +Ri_{5}\sin\left(-12\omega t+\theta_{5}\right)+7\omega L_{d}i_{7d}+Ri_{7q}+6\omega L_{q}i_{d7}+\ldots \end{array} \end{array}\right] \end{array} \end{array}\right.$$

From Equation (10), we can see that in the 5*d*-*q* coordinate system, only the 5th-order current is the DC component, while the rest are AC components. A low-pass filter can be used to extract the 5th harmonic component. The 7th harmonic component can also be extracted similarly [37]. Meanwhile, according to the tracking characteristics of the PI controller without static error and, given *i*_d5_ = 0, *i*_q5_ = 0, *i*_d7_ = 0, and *i*_q7_ = 0, the current harmonics pass through the PI regulator, and then the coordinates of 5*d*-*q* and 7*d*-*q* are transformed into a *d*-*q* coordinate system through Equation (15). Then, the corresponding voltage compensation can be obtained [36,37]:

(15) $$\left\{\begin{array}{c} T_{5dq\to 1dq}=\left[\begin{array}{cc} \cos(-6\theta ) & -\sin(-6\theta )\\ \sin(-6\theta ) & \cos(-6\theta ) \end{array}\right]\\ T_{7dq\to 1dq}=\left[\begin{array}{cc} \cos(6\theta ) & -\sin(6\theta )\\ \sin(6\theta ) & \cos(6\theta ) \end{array}\right] \end{array}\right.$$

(16) $$\left[\begin{array}{c} u_{d}\\ u_{q} \end{array}\right]=\left\{\begin{array}{c} \begin{array}{c} 5dq:\\ \left[\begin{array}{c} 5\omega L_{q}i_{q5}+Ri_{d5}+6\omega L_{d}i_{q5}\\ -5\omega L_{d}i_{d5}+Ri_{q5}-6\omega L_{q}i_{d5} \end{array}\right] \end{array}\\ \begin{array}{c} 7dq:\\ \left[\begin{array}{c} -7\omega L_{q}i_{q7}+Ri_{7d}-6\omega L_{d}i_{7}\\ 7\omega L_{d}i_{7d}+Ri_{7q}+6\omega L_{q}i_{d7} \end{array}\right] \end{array} \end{array}\right.$$

**Figure 12:**
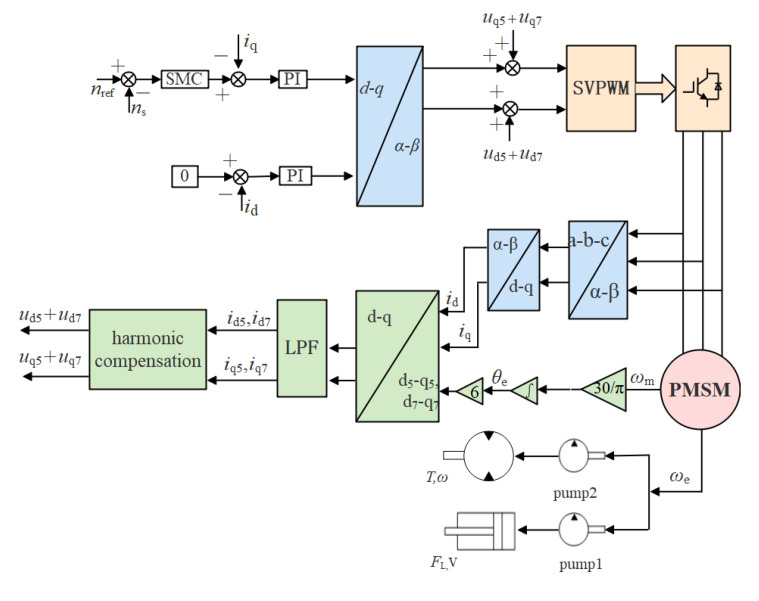
Functional block diagram of pump controlling the hydraulic system based on the CCSSMC-HS.

To enhance the robustness of the control system, we propose a cooperative control algorithm that combines the SMC and harmonic suppression compensation. The principle of collaborative control system based on SMC and harmonic suppression (CCSSMC-HS) is shown in Figure 12.

### 5.2. Control Method Improvement Results and Analysis

**Figure 13:**
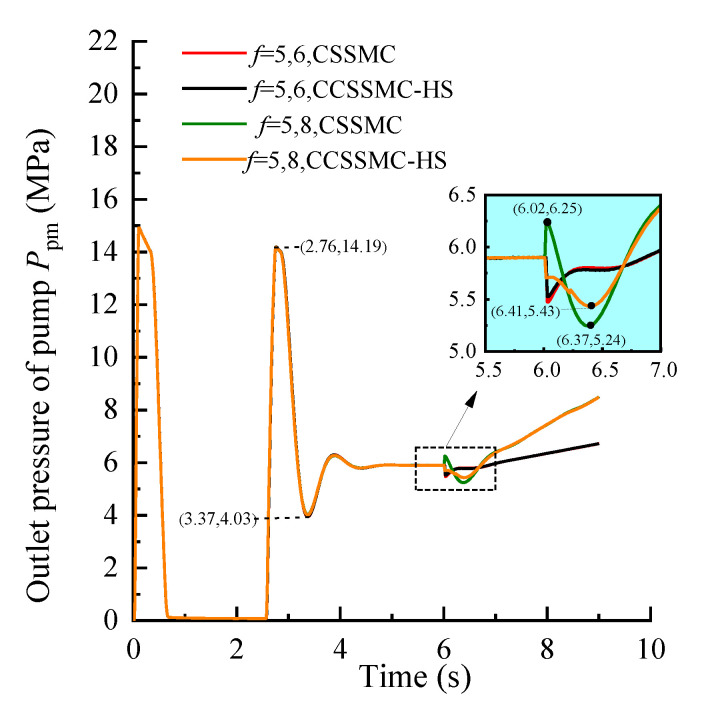
Variation curve of the hydraulic pump outlet pressure based on the CSSMC and CSSMC-HS.

**Figure 14:**
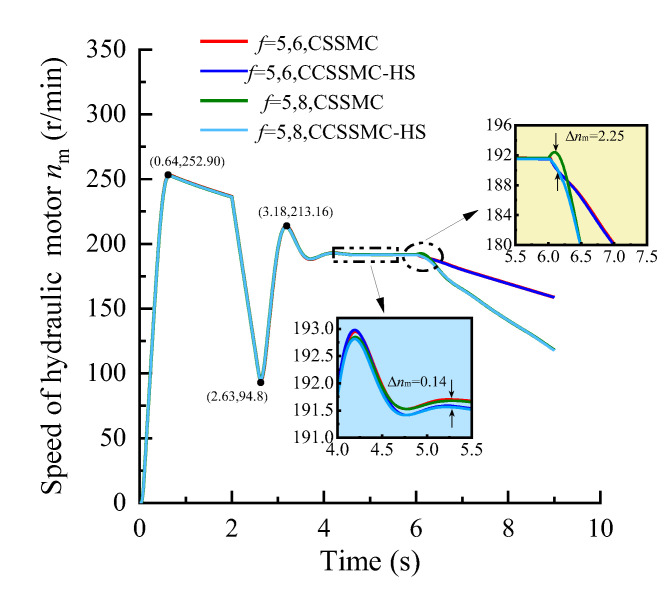
Variation curve of the hydraulic motor speed based on the CSSMC and CSSMC-HS.

**Figure 15:**
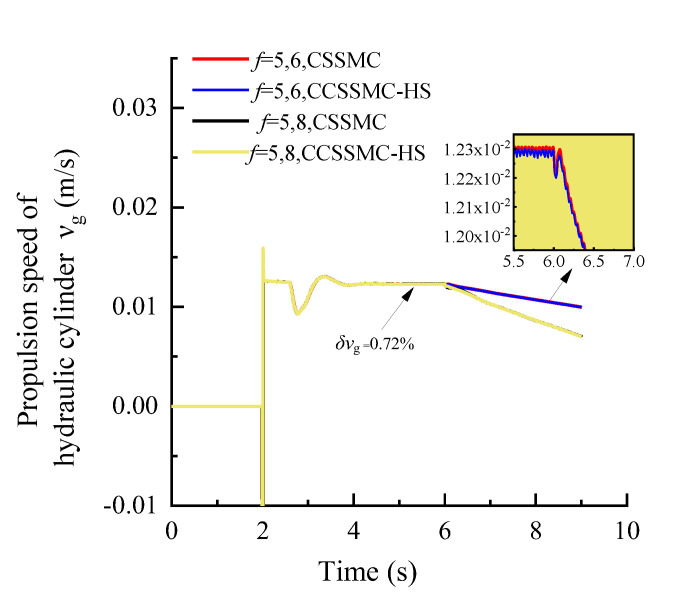
Variation curve of the hydraulic cylinder speed based on the CSSMC and CSSMC-HS.

**Figure 16:**
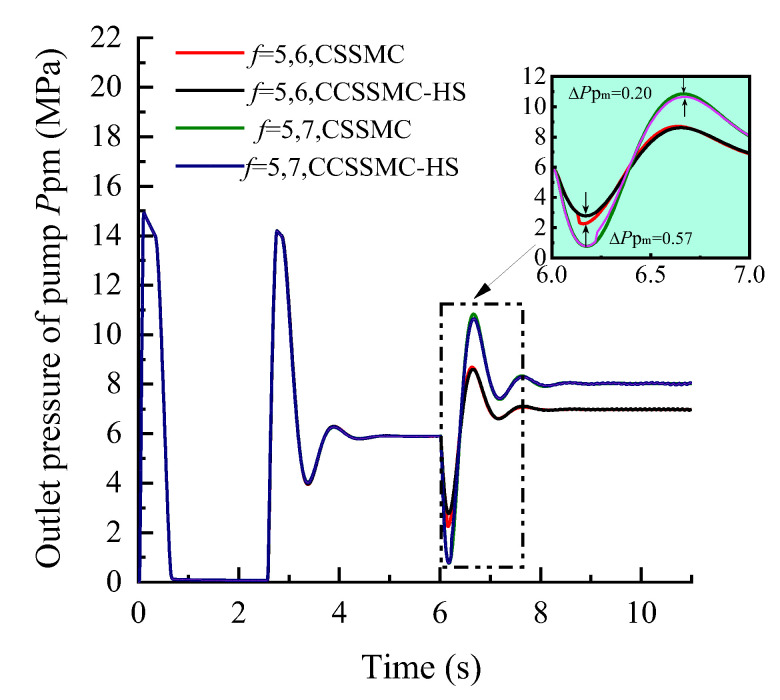
Variation curve of the hydraulic pump outlet pressure based on the CSSMC and CSSMC-HS.

**Figure 17:**
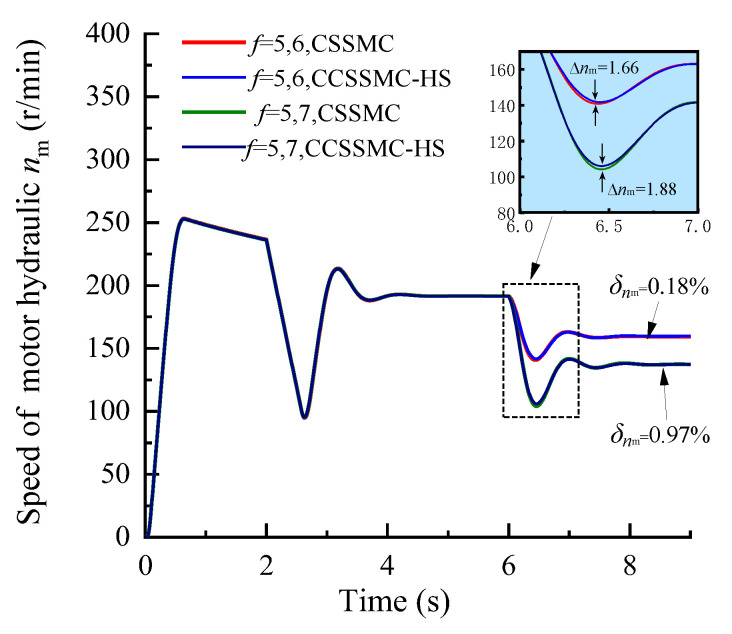
Variation curve of the hydraulic motor speed based on the CSSMC and CSSMC-HS.

**Figure 18:**
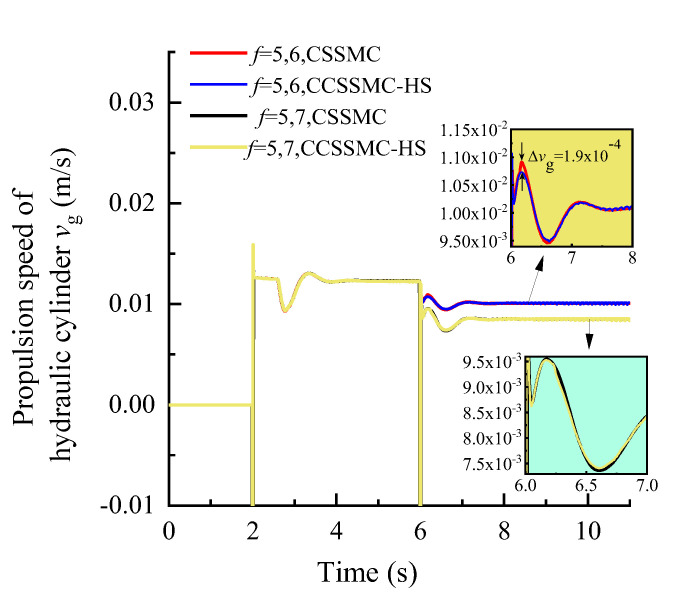
Variation curve of the hydraulic cylinder speed based on the CSSMC and CSSMC-HS.

In order to compare and analyze the performance of the control strategy based on the CCSSMC-HS, the experimental simulation parameters and process are kept the same as those used and completed in the previous section. The results are shown in Figure 13, Figure 14, Figure 15, Figure 16, Figure 17, Figure 18.

Figure 13, Figure 14, Figure 15 show that the change curve of each physical quantity of the hydraulic system based on the CSSMC-HS coincides with the curve based on the CSSMC. The relative deviation between the time when they enter the steady state and the pulsation coefficient under the steady state are both less than 2%. While the pump controlling the hydraulic system was based on the CSSMC (the rock hardness factor gradually changed from *f* = 5 to *f* = 8), the hydraulic pump outlet pressure overshoot was reduced from 11.19% to 7.97% compared with that before the optimization, which reduced the impact of the load change on the system stability.

The change law of each physical quantity of the hydraulic system based on the CSSMC-HS in Figure 16, Figure 17, Figure 18 is the same as that shown in Figure 13, Figure 14, Figure 15. When the rock stiffness factor increased suddenly from *f* = 5 to *f* = 6, the pressure overshoot at the outlet of the hydraulic pump of the pump-controlled motor system was reduced from 61.19% to 52.88%, compared with that before the optimization. The reason for this is that as the PMSM operates at a high speed, there is a sudden change in the load torque and the influence of the harmonic current in the system is significantly enhanced, which in turn decreases the rigidity of the hydraulic system driven by the motor. For the CSSMC-HS, the harmonic components are suppressed and compensated for; hence, the rigidity of the hydraulic system driven by the motor is enhanced. That is, the proposed vector control strategy based on the CSSMC-HS can effectively reduce the impact of load disturbance on the system’s performance.

## 6. Conclusions

(1) Following the pump control mechanism of PMSM and the system theory of interaction between anchoring equipment and coal rocks, a mathematical model of the power control system of step-type anchoring equipment was established, and the influence of coal rock hardness factor *f* on the stability of the power system of the anchoring equipment was analyzed.
(2) A control strategy applicable to the drive system of walking anchoring equipment based on the CSSMC was also proposed. Compared with the CSPI, the dynamic characteristics of the hydraulic pump outlet pressure, hydraulic motor output speed, and pump-controlled hydraulic cylinder advance speed in the pump-controlled motor hydraulic circuit of this control method were greatly improved, while the maximum pulsation coefficient of each variable after the system entered the steady state was less than 3%.
(3) Furthermore, the CSSMC was optimized by suppressing and compensating the harmonic components of PMSM in the power system. For the CSSMC-HS, when the coefficient of coal and rock hardness in action gradually changed from *f* = 5 to *f* = 8 and abruptly changed from *f* = 5 to *f* = 6, the hydraulic pump outlet pressure overshoot fell from 11.19% to 7.97% and from 61.19% to 52.88%, respectively, compared to the levels prior to optimization. This optimized control strategy was therefore able to mitigate the impact of load disturbance on the system’s performance.

## Figures and Tables

**Table 1:** Basic parameters of PMSM and the hydraulic system [27,30].

Symbol	Physical Significance	Value
*L*_q_ (mH)	*q*-axis inductance	50
*L*_d_ (mH)	*d*-axis inductance	50
*R* (Ω)	stator winding resistance	1.3
*ψ*_f_ (Wb)	motor rotor flux	1
*J*_T_ (kg·m^2^)	moment of inertia	0.09
*B*_T_ (N·m·s)	damping coefficient	0.0008
*P*_n_	number of motor rotor pole pairs	2
*t*_d_ (μs)	dead time	3
*u*_d_ (V)	pressure drop of pipe resistance	1
*D*p_g_ (m^3^/rad)	displacement of pump1	2.5 × 10^−6^
*D*p_m_ (m^3^/rad)	displacement of pump2	4 × 10^−5^
*η*_pm_	mechanical efficiency of pump	0.95
*β*_b_(N/m^2^)	bulk elastic modulus of oil in a closed chamber	9.0 × 10^8^
*D*_m_ (m^3^/rad)	displacement of motor	8 × 10^−5^
*J*_m_ (kg·m^2^)	equivalent moment of inertia of motor output shaft	3
*d*_1_ (mm)	diameter of rod cavity of hydraulic cylinder	40
*d*_2_ (mm)	diameter of rodless cavity of hydraulic cylinder	20
*D* (mm)	diameter of outside the drill	27
*B*_g_	damping coefficient of hydraulic cylinder	1000
*s* (m)	stroke of hydraulic cylinders	2
*k*_p_	scale factor of PI	250
*k*_i_	integral time constant of PI	6500
*c*	sliding surface parameter of SMC	2
*ε*	the system overcomes the perturbation and external interference parameter of SMC	171
*q*	approaching speed parameter of SMC	55

**Table 2:** Pulsation analysis of the CSPI and CSSMC.

Physical Variable	*f* = 5		*f* = 6		*f* = 7	
	CSPI	CSSMC	CSPI	CSSMC	CSPI	CSSMC
the pulsation coefficient of the outlet pressure of pump	6.46%	2.10%	5.87%	0.39%	4.03%	0.98%
the pulsation coefficient of the hydraulic motor speed	1.12%	0.69%	1.32%	0.18%	4.72%	0.97%
the pulsation coefficient of the hydraulic cylinder speed	18.18%	0.72%	17.02%	0.31%	44.09%	0.50%

## Data Availability

Not applicable.
